# Pressure-Dependent Structural and Luminescence Properties of 1-(Pyren-1-yl)but-2-yn-1-one

**DOI:** 10.3390/molecules24061107

**Published:** 2019-03-20

**Authors:** Anna Makal, Joanna Krzeszczakowska, Roman Gajda

**Affiliations:** Faculty of Chemistry, Biological and Chemical Research Centre, University of Warsaw, ul. Zwirki i Wigury 101, 02-096 Warsaw, Poland; joannakrzeszczakowska@gmail.com (J.K.); romanbg@chem.uw.edu.pl (R.G.)

**Keywords:** polynuclear aromatic hydrocarbon, π-stacking, high-pressure, diamond anvil cell, X-ray structure determination, intermolecular interaction energy, luminescence

## Abstract

The crystal structure of 1-(pyren-1-yl)but-2-yn-1-one (1a, a polynuclear aromatic hydrocarbon displaying enhanced luminescence in the solid state, has been re-determined at several pressures ranging from atmospheric up to 3 GPa using a Diamond Anvil Cell (DAC). These experiments were augmented by periodic DFT calculations at pressures up to 4.4 GPa. UV-Vis fluorescence of 1a at non-ambient pressures has also been investigated. The crystal structure consists of infinite π-stacks of anti-parallel 1a molecules with discernible dimers, which may exemplify aggregates formed by pyrene derivatives in solution and thin films, and is predominantly stabilized by dispersion. The average inter-planar distance between individual molecules within π-stacks decreases with pressure in the investigated range. This results in piezochromic properties of 1a: a red-shift of sample color, as well as a bathochromic shift of fluorescence with pressure (by ca. 100 nm at 3.5 GPa). Two-component fluorescence spectra support the hypothesis that at least two types of excimers are involved in the electronic excitation processes in crystalline 1a.

## 1. Introduction

Polycyclic Aromatic Hydrocarbons (PAHs) are objects of interest due to their potential use in optoelectronics [1]. Among them, pyrene derivatives show several interesting applications as electronic components [2,3], charge transfer materials [4], or luminescent biomarkers [5,6]. As a rule, these compounds show high sensitivity to the environment [7,8,9], manifested for instance in their varying spectroscopic properties in different solvents or when attached to biological macromolecules. In particular, the molecular environment of a PAH encased in an aggregate will influence the physicochemistry of such a supramolecular construct. Indeed, the ability of PAHs to form aggregates, ranging from π-stacked dimers to more intricate arrangements [10,11] in solution, as well as thin films and in the solid state, is vital to many of their applications.

In many instances, the aggregation of aromatic moieties in concentrated solution or in a crystal results in luminescence quenching. This appears to be the case with so-called H-aggregates [12], i.e., parallel stacks of aromatic entities with significant π-orbital overlap [13,14]. However, there are also cases where certain modes of aggregation in the solid state enhanced the luminescence of such compounds [11,15].

**Figure 1:**
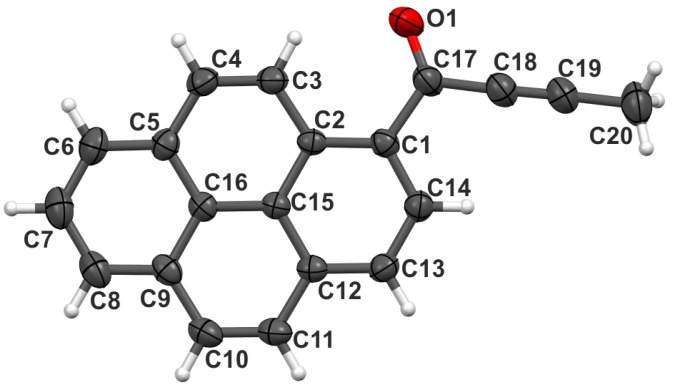
The molecule of compound 1a at ambient pressure and room temperature. Atomic displacement parameters represented at the 50% probability level. Hydrogen atom numbers are the same as the numbers of connected C atoms.

The compound proposed for investigation here, 1-(pyren-1-yl)but-2-yn-1-one (Figure 1), further denoted as 1a, is a fine example of enhanced fluorescence in the solid state [16], with a relatively long lifetime of the excited state. Time-resolved spectroscopy suggested that such a long-lived excited state is indeed the result of excimer formation.

As the crystal structure of 1a consists of infinite π-stacks, the distance between the pyrene π-systems may be expected to influence the fluorescent properties of the compound. Changing this distance in a controllable and reversible way should significantly tune the spectroscopic properties of this material and shed some light on the mechanism of its luminescence.

One way to gain control over intermolecular distances in a crystal is the application of hydrostatic pressure. Studying rearrangements occurring in the crystal structures under pressure can also be the means of ascertaining the importance and relative hierarchy of intermolecular interactions [17,18]. Structural analysis at high pressure can therefore help explain the physicochemical properties of materials thus analyzed, as presented, e.g., in the review works by Parsons [19], Fanetti [18], or Zakharov [20]. In particular, a constricted crystalline environment may effectively mimic the presence of certain aggregates, which under standard conditions would be extremely short-lived and therefore hard to trace.

Interestingly, PAH-s and especially pyrene derivatives have not been so far extensively studied by X-ray diffraction at high pressure. The works of Fabbiani et al. [21,22] described the structure evolution and phase transitions in naphthalene, anthracene, and unsubstituted pyrene recrystallized under pressure, at pressures up to 2.1 GPa. The literature cited therein suggested that carbonization of crystalline naphthalene may occur at a pressure as low as 2.5 GPa. On the other hand, the X-ray diffraction study by Capitani [23] carried out up to 25 GPa showed that phenanthrene remained crystalline up to ca. 20 GPa, undergoing phase transition around 8 GPa. A recent paper by Chanyshev et al. [24] dealt with high-pressure and high-temperature-induced structural changes in benzene. Apart from that, a short note on the structure of benzo[a]pyrene [25] under pressure is available. In a wider perspective, a high-pressure study of organic conductor rubrene [26] showed that pressure-induced molecular rearrangement resulted in a phase transition and a loss of conducting properties of that material above 6 GPa.

Here, we present the analysis of the structural changes introduced in the crystal lattice of 1a by increased pressure. We aim at comparing the experimental structures with the results of theoretical calculations and at linking structural changes to reorganization of electronic levels. In addition, the role of dispersive π⋯π interactions in the stabilization of its crystal structure is being highlighted.

## 2. Results and Discussion

### 2.1. Structure at Ambient Pressure

The crystal structure of 1a has already been determined at 100 K [16]. In order to have a reference for the high-pressure studies, the structure was re-determined at room temperature and ambient pressure. A series of short single-crystal X-ray diffraction experiments was also conducted in order to ensure that no phase transitions took place between room temperature and 100 K.

Apart from thermal expansion of the unit cell and larger Atomic Displacement Parameters (ADPs), the current structure is the same as the one originally determined. There was a single molecule of 1a in an asymmetric unit, with the pyrene moiety almost exactly planar (the average planes of the terminal benzene rings are inclined at an angle of 3.3(3)${}^{\circ }$), and the carbonyl substituent rotated out of the average pyrene plane by only 8.0(2)${}^{\circ }$. A relatively strong intramolecular C3–H3⋯O1 hydrogen bond [16] may be responsible for the almost perfect planarity of the molecule.

**Figure 2:**
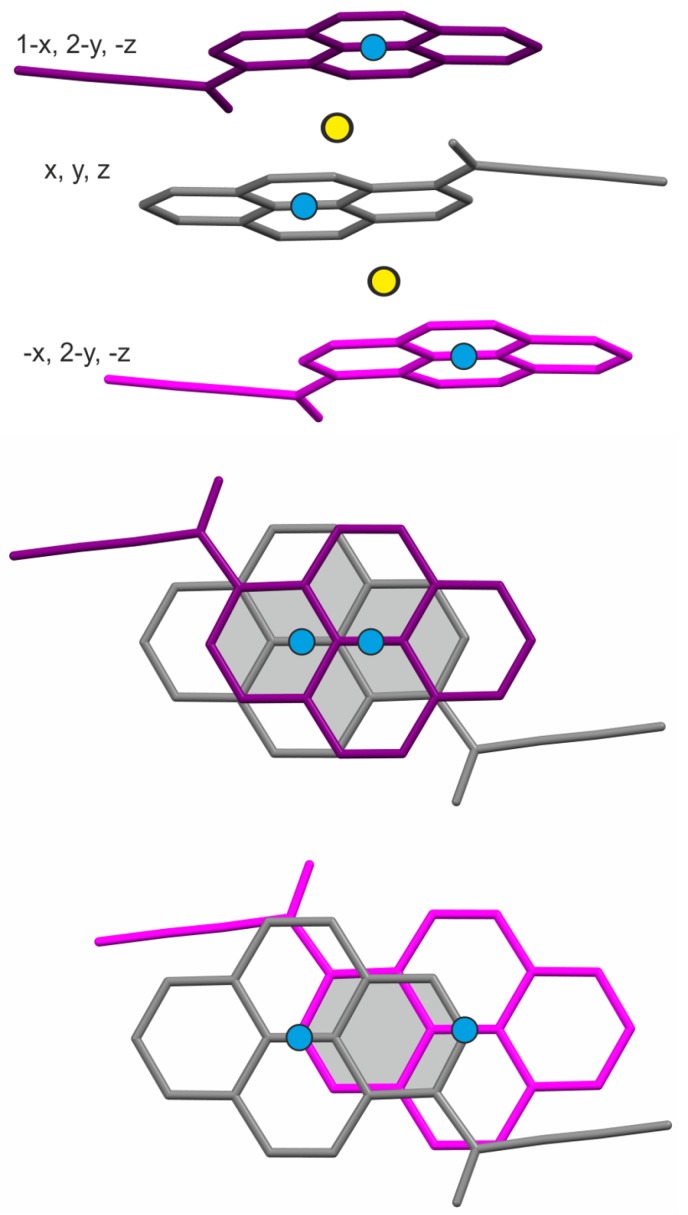
Top: a fragment of the infinite π-stack in the structure of compound 1a viewed along [001]; crystallographic centers of symmetry indicated as yellow dots; the independent molecule of 1a in the asymmetric unit represented in grey, the two closest neighbors of 1a in a stack represented in purple and magenta, with symmetry codes of the molecules on the left; the centers of each pyrene moiety represented as blue dots. Middle and bottom-views perpendicular to the average pyrene planes, roughly along [100], illustrating the vertical overlap of pyrene moieties and the lateral shifts of their centers; the longer interplanar distance (purple) is associated with larger vertical overlap of pyrene fragments.

The molecules form infinite π-stacks in [100] direction, where stacked moieties are related by crystallographic inversion centers (Figure 2). The shortest distances between the average pyrene planes within a stack are 3.464(4) Å (“grey” and “purple” molecules in Figure 2) and 3.401(3) Å (grey and pink molecules in Figure 2). The angle at which the pyrene moiety is inclined with respect to the stacking direction is 75.5(5)${}^{\circ }$. Viewed from above (ca. along [100]), molecules in π-stacks show significant horizontal overlap of the pyrene fragments (Figure 2). In particular, the molecules with a slightly longer interplanar distance show significant vertical overlap of pyrene fragments. A π-stack can therefore be considered as a “stack of dimers”. Such stacking may enable the effective formation or stabilization of a few imaginable excited multimers in the electronic excitation process.

**Figure 3:**
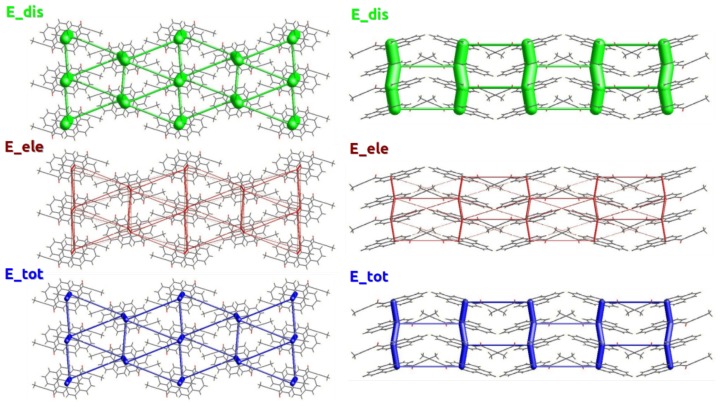
Energy frameworks in the crystal structure of 1a. Green, red, and blue bars represent dispersion, electrostatic, and total interaction energies accordingly. The thickest bars among the total intermolecular interaction energies correspond to energy of −63.8 kJ/mol and thin bars to energies within the range −10 to −12 kJ/mol. Energies with a magnitude less than −10 kJ/mol have been omitted. Views along the π-stacking direction [100] (**left**) and along [001] (**right**).

The inter-stack interactions consist of long C–H⋯O H-bonds extending in roughly [001] direction and numerous H⋯H contacts in other directions (Figure 3). The structure may be considered as a collection of well-separated stacks. Referring to the work of Desiraju and Gavezzotti [10], the structure of 1a can be classified as γ-type, although it does not fulfill the criteria for the length of the shortest cell axis (i.e., **a** is longer than 5.4 Å).

### 2.2. Energy Frameworks

The importance of the dispersive π⋯π interactions in the stabilization of the crystal structure of 1a is best highlighted by visualization of its energy framework (Figure 3). It is immediately seen that the dispersive component is the most important part of the total interaction energy of molecules within the π-stack, as well as in inter-stack interactions. There is no interatomic interaction present, in which the electrostatic term would play a dominant role (Table 1). The total intermolecular interaction energy of the stacked molecules is ca. −62 kJ/mol, comparable with strong H-bonding. Notably, the values of lattice and intermolecular interaction energies at ambient pressure conform well to the CrystalExplorer estimates. It is also clear that the interactions between the stacks are at least twice weaker than inter-stack ones. The next strongest interaction in the crystal lattice, involving the weak C–H⋯O bonds, results in intermolecular interactions no stronger than −28 kJ/mol. The energy framework viewed along the [100] direction is a typical [27] hexagonal lattice. With such pronounced differences in the strength of intermolecular interactions in the 1a crystal, it can be expected to display significant anisotropy when subject to physical stimuli.

### 2.3. Unit Cell Changes and Strain Analysis at Non-Ambient Conditions

**Figure 4:**
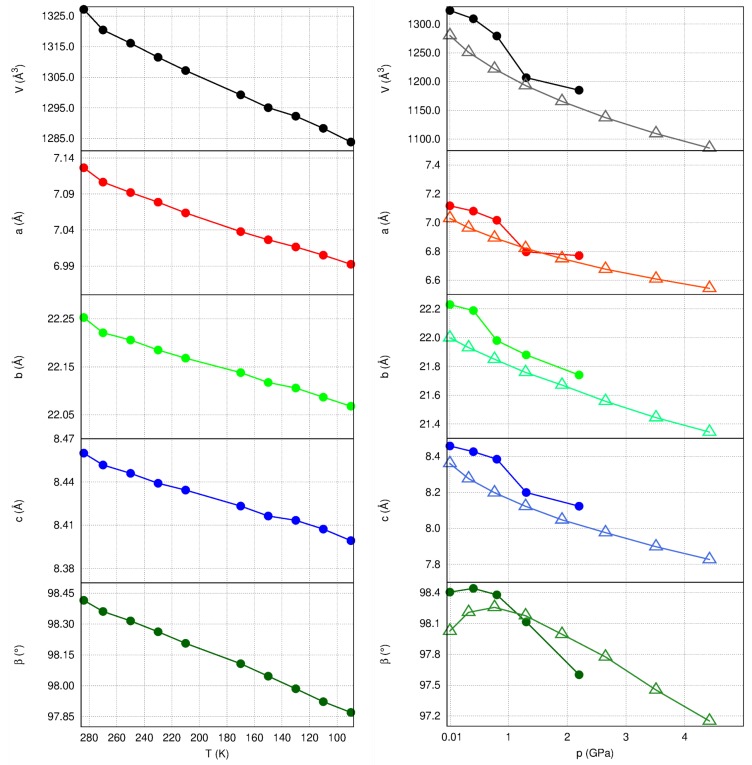
Unit cell parameter changes with temperature (**left**) and pressure (**right**). Experimental results represented by filled markers, results of theoretical calculations—as empty ones.

Changes in the unit cell parameters of 1a induced by increased pressure or varying temperature are presented in Figure 4. There was a uniform tendency of all variables, but the β angle to shrink monotonically with increased pressure or lowered temperature. Experimental values tended to be slightly higher than theoretical predictions for the increasing pressure series. The discrepancies did not exceed 4% of the values at atmospheric pressure. Up to 4.5 GPa, there were no indications of possible phase transitions.

Analysis of the principal strain axes, which can be performed using the PASCal server [29], allows quantifying the compressibility of a material and identifying the directions, which are the most important mechanically in the crystal structure. These may, or may not, coincide with crystallographic directions in the low-symmetry systems, but can be effectively linked to the most important intermolecular interactions. A summary of the strain tensor analysis for 1a is given in Table 2.

Interestingly enough, 1a showed the greatest compressibility in the direction of π-stacks (approaching 5% at 2.2 GPa according to experiment). This is also the direction of the largest thermal expansion. Such a coincidence is in accordance with the opposite behavior of organic materials in response to an increase in either temperature or pressure [30,31]. On the other hand, the direction in which, according to theoretical predictions, a crystal is the least compressible is roughly along the direction of the acetylene fragment, in which intermolecular interactions consist mainly of H⋯H contacts. These are not very stabilizing even at ambient conditions (see Table 1, the molecules related by −x, y + 1/2, −z + 1/2 symmetry) and may be expected to become repulsive very quickly with shortening intermolecular distances.

Thermal expansion is the smallest in roughly [203] direction, i.e., in the direction in which the C–H⋯O H-bonds are present. According to estimates of intermolecular interaction energies, these directional interactions do not effectively withstand compression, but they seem to act as “clamps” in the case of intensifying molecular vibrations.

The bulk modulus $B_{0}$ of 1a, estimated from theoretical calculations using a third order Birch–Murnaghan equation of state [32], equals 14.3(4) GPa, and its derivative B’ = 8.1(4). $B_{0}\approx$ estimated by a second-order Birch-Murnaghan equation of state for the few experimental observations is 13(3) GPa. The value of $B_{0}$ was higher than reported for other PAHs investigated at high pressure [23,26] (i.e., 5.7 GPa for phenanthrene, 8.2 GPa for rubrene, or 9.6 for pentacene), indicating that crystals of 1a are in fact slightly harder than the above-cited polyaromatic materials, though still relatively soft.

### 2.4. Structural Changes at Non-Ambient Conditions: Experiment and Theory

There were no spectacular changes in the molecular structure upon increased pressure. Bond lengths obtained experimentally at various pressures were the same within experimental uncertainties (Table S2 in the Supporting Information). In the case of theoretical results, there was a tendency for most of them to shorten with pressure, but the shortening did not exceed 0.4% of a bond-length at ambient pressure. The only exception was the carbonyl C=O bond: according to theoretical calculations, its length tends to increase with pressure, possibly due to the increased role of C–H⋯O H-bonds.

**Figure 5:**
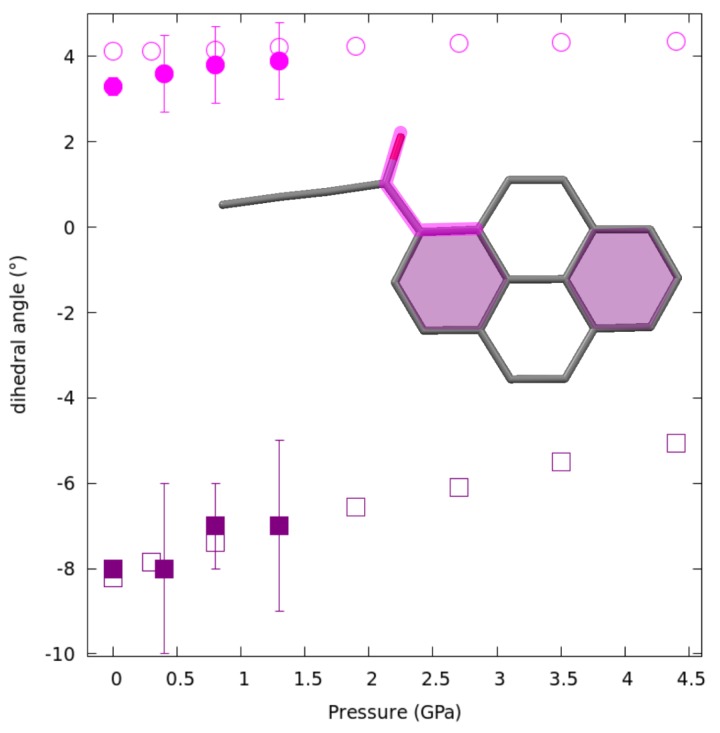
Variation of the C=O out-of-plane rotation (purple squares) and the angle between terminal benzene rings (magenta circles) with pressure. Experimental results represented by filled markers with uncertainties, results of theoretical calculations by empty ones.

There was a tendency for the carbonyl moiety to become even more co-planar with pyrene (O1-C17-C1-C2 dihedral in Figure 5) and for the pyrene moiety to get slightly more bent along the short pyrene axis.

**Figure 6:**
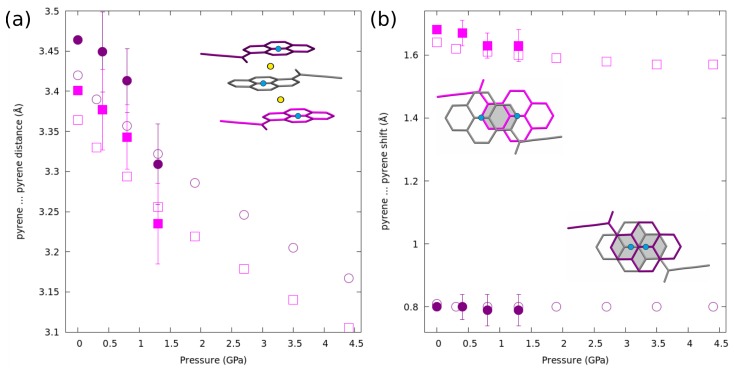
Variation of the (**a**) inter-planar distances in a π-stack with pressure; the purple-grey pair represents the “dimer” within the stack; (**b**) lateral shifts involving 1a molecules within a “dimer” (purple circles) and molecules from consecutive dimers (magenta squares) with pressure. The centers of each pyrene moiety represented as blue dots, crystallographic centers of symmetry—as yellow dots. Experimental results represented by filled markers with uncertainties, results of theoretical calculations by empty ones.

However, pronounced structural changes occurred in the case of intermolecular distances, as shown by both experiments and theory. The large compressibility of 1a in the [100] direction results from the pyrene moieties within π-stacks being pushed closer together (Figure 6a and Table S2 in the Supporting Information). The decrease was small, but statistically significant, reaching 0.12 Å at 1.3 GPa according to experiment and over 0.25 Å at 4.4 GPa as predicted by theoretical calculations. A similar, but not so spectacular trend can be observed for the C⋯O in the intermolecular H-bonds (Figure S2 in the Supporting Information). Interestingly, the inclination of the pyrene plane with respect to the stacking direction did not change with pressure, remaining always ≈75${}^{\circ }$ (Table S2 in the Supporting Information).

The compression of the 1a reveals the different response of the molecules within dimers and from two consecutive dimers in a stack. The relative lateral shift between two molecules is defined here as half of the distance between the center of one pyrene moiety and an orthogonal projection of the center of the next one. Within a “dimer”, this shift remains constant, indicating that such a dimer is a conserved synthon in this crystal structure. In the case of molecules from two consecutive dimers, a small decrease in the lateral shift of each pyrene molecules can be observed (Figure 6b).

### 2.5. Theoretical Calculations

As presented above, molecular and crystal structures are in very good agreement with the experimental data. The only noticeable differences were the C–H bond distances: in the case of experimental models, these were intrinsically shorter, constrained to SHELXL standard lengths [33] (Figure S1 in Supporting Information), while the optimized distances were close to the average neutron values [34]. The theoretical results can therefore be directly linked with the experimental outcomes.

The lattice-stabilizing energy decreased with increased pressure, becoming twice less negative at 4.4 GPa than at no external pressure. The most important in-stack interactions became less stabilizing fast, showing interaction energies of only −3.58 kJ/mol at 4.4 GPa (Table 3). The trend suggests that such an interaction would become repulsive at around 5 GPa.

1a is a luminescent material in bulk and can be expected to approach a semiconducting state due to the extensive π-stacking in the crystal. Here, we decided to analyze its band structure and how it changes when molecular π-orbitals are pushed closer together with pressure.

According to the calculated band structure, 1a in standard conditions can indeed be classified as an indirect semiconductor, with the band gap of 2.6 eV (0.093 Ha), slightly smaller than the band gap of ZnO (0.12 Ha) and only slightly larger than the band gap of crystalline silicon (0.04 Ha).

Analogous to the calculations for isolated molecules, where the HOMO-LUMO gap can be related to spectroscopic properties, the energy band gap in the solid state may be translated into the predicted UV-Vis absorption maximum. The calculated band gap span of 1a indicates $\lambda_{max}^{absorption}$ ca. 489 nm (Table S3 in the Supporting Information), about 30 nm longer than observed for a powder sample [16]. The difference from the previous results may reflect the inadequacies of the assumed calculation method or different calibration of the equipment used in the compared experiments.

**Figure 7:**
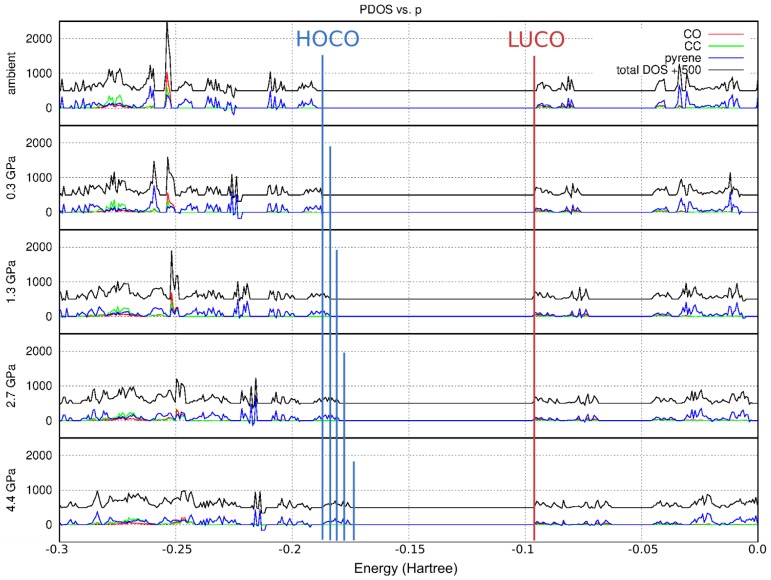
Electronic Partial Density Of State (PDOS) plots in the vicinity of the band gap theoretically calculated for crystalline 1a. Shrinking of the band gap with pressure is clearly visible. The highest occupied energy band is dominated by contributions from the pyrene moiety (blue), while a small contribution from the C=O (red) and C≡C (green) fragments is visible in addition to that of pyrene at the lowest unoccupied energy levels.

A Partial Density Of State (PDOS) plot is another way of describing the electronic structure of the crystalline system. In particular, it is a quick way to assess which molecular fragments contribute most to this structure at a certain energy level, and which are therefore the most important in electronic excitation processes. In the case of 1a, the Highest Occupied Crystalline Orbitals (HOCO) form a well-separated band and are dominated by the contribution from the pyrene moiety (Figure 7). On the other hand, the Lowest Unoccupied Crystal Orbitals (LOCO) show contributions from C=O and C≡C fragments, but still, the pyrene moiety plays an important role.

**Figure 8:**
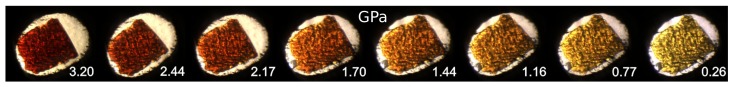
Microscopy images of a 1a crystal in a Diamond Anvil Cell (DAC) (gasket diameter ≈400 μm, a small ruby chip is visible at the lower-left corner). Gradual darkening of the sample with pressure indicates a red-shift in the UV-Vis absorption spectrum. The crystal has been cycled from low to high pressure and back several (six) times, with repeatable results.

The bands broadened with pressure (Figure 7). As the pressure increased, the band gap decreased, which agrees with the red-shift of the absorption energy (to 564 nm, or 0.08 Ha at ca. 4 GPa) and which is also reflected in the sample’s spectacular color change from light yellow at standard pressure to dark red at 3.5 GPa (Figure 8). The decrease of the band gap resulted uniformly from the energy levels of HOCO becoming less negative with pressure (destabilized by closer proximity of molecules within π-stacks). The energies of unoccupied levels remained completely unaffected.

### 2.6. Luminescence Changes

**Figure 9:**
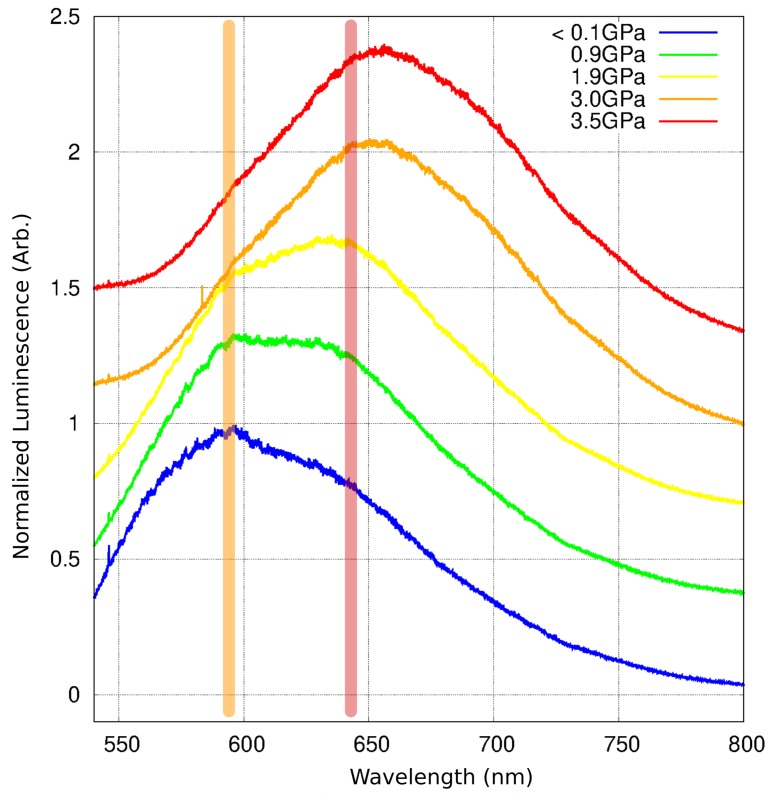
Luminescence spectra of 1a at selected pressures. Excitation wavelength: 532 nm. The spectra are normalized and offset along the y-axis for clarity. The orange and red bars indicate the emission maxima assigned to the initial small excimers (possibly dimers) and higher multimers accordingly.

The observed piezochromism clearly reflects the shift in maximum wavelength of absorbed light. It is accompanied by a significant shift in the averaged fluorescence maxima upon increasing pressure. While at atmospheric pressure, the compound displayed a broad yellow fluorescence band ($\lambda_{max}$ at 597 nm, again about 30 nm longer than the value previously recorded for a powder sample at ambient conditions [16]), the emission became significantly red-shifted with increased pressure, reaching 656 nm at 3.3 GPa (Figure 9).

**Figure 10:**
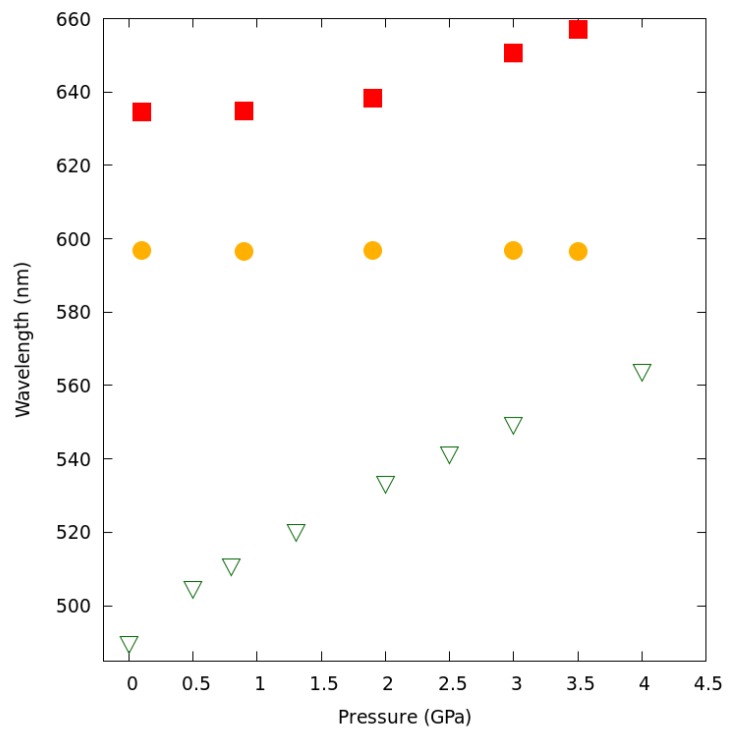
The variation of the theoretically-predicted wavelength of maximum absorption (green triangles), experimentally-observed $\lambda_{max}^{emission}$ attributed to small aggregates (the orange band represented as orange circles), and the experimentally-observed $\lambda_{max}^{emission}$ attributed to larger aggregates or multimers (the red band represented as red squares). The latter shows small variation with pressure above 2.0 GPa.

Notably, the emission band at low pressure appears to consist of two components: one with maximum ca. 597 nm (orange band) and another, weaker, at about 635 nm (red band). As the pressure increases, the contribution of the red-band becomes more prominent, and at above 3.0 GPa, the contribution from the orange band is hardly noticeable. While the position of the orange band appears to be constant with pressure, the red-band maximum shows a small red-shift with pressure above 2.0 GPa (Figure 10).

The former time-resolved fluorescence study [16] performed on a powdered sample of 1a for emission wavelengths of 540 nm and 640 nm showed very different rise and decay times at these emission wavelengths. Specifically, a very short decay-time for the 540 nm emission was almost the same as the rise-time of the emission at 640 nm. A short decay-time in the blue-edge of the spectrum, corresponding to a short rise-time in the red part of the spectrum, was considered as a signature of excimer formation, which occurred within the crystal with a fast kinetic rate. Small-size multimers (for instance dimers) were expected to form almost immediately, and then disappear, being incorporated into more complicated excited multimers.

The $\lambda_{max}^{em}$ of the currently-observed orange band (ca. 597 nm) is already far from the emission maximum of a 1a monomer, as previously recorded in a diluted CHCl${}_{3}$ solution (449 nm [16]). A tentative conclusion would be that this band originates from electronic excitations occurring in a small aggregate of 1a, possibly the very dimer that can be distinguished within the crystal structure. A slight difference in interplanar distances and the interaction energies between molecules in a π-stack would support such an assumption. The red band could than be assumed to originate from excitations involving more complicated multimers, in accordance with the former time-resolved study. As the increased pressure forces molecules closer together, the formation of the latter, many-component multimers can occur almost instantly, and emission from such multimers would dominate the spectrum. It must be stated that more definite conclusions could only be made based on a pressure-dependent time-resolved luminescence study, which would allow comparing luminescence decay-times of the orange and red bands.

## 3. Materials and Methods

### 3.1. Crystallization

The compound was synthesized according to a procedure described elsewhere [16]. Single crystals of 1a were obtained by slow diffusion of n-pentane into the saturated chloroform solution of the compound. The crystals were large yellow oblong prisms or thick plates with well-formed faces. A single specimen was used for the structure re-determination at room temperature and the multi-temperature diffraction study.

### 3.2. Sample Preparation for High Pressure Experiments

A single crystal of a suitable size was obtained by cutting down a selected large specimen to appropriate thickness. It was then placed in an Almax Plate Diamond Anvil Cell (DAC) of the modified Merrill and Bassett design [36] together with a small piece of ruby, the latter serving as an internal pressure calibrant. The DAC was equipped with 0.75-mm culet diamonds and a steel gasket of an initial thickness of 0.3 mm and a 0.5-mm gasket hole. The DAC’s nominal maximal opening angle was 42${}^{\circ }$. A Paratone oil was used as the pressure-transferring medium. The choice of oil as the Pressure-Transmitting Medium (PTM) was dictated by the fact that 1a is soluble in a more suitable MeOH/EtOH mixture, but upon recrystallization, attempts at low pressures yield crystals of very inferior quality.

At each investigated pressure point, the pressure within the gasket hole was estimated by the ruby fluorescence method [37,38] using an Almax Optiprexx PLS spectrometer, affording the precision of 0.1 GPa.

### 3.3. Ambient Pressure X-Ray Data Collection and Refinement

The reference X-ray crystal structure of 1a at room temperature was obtained using the data collected on the KAPPA APEX-II Ultra 4-circle diffractometer, with a molybdenum rotating anode as an X-ray source and multi-layer focusing mirrors. A single specimen was mounted on top of a thin glass capillary with epoxy resin. The data were collected with Bruker APEX-II software (Bruker AXS Inc., Madison, WI, USA) [39], integrated using the Bruker SAINT software package (Bruker AXS Inc., Madison, WI, USA) [40], and corrected for absorption effects using the multi-scan method (SADABS [41]).

The structure was solved by SHELXT [33] and then refined by SHELXL [42], both algorithms adopted in the Olex2 graphical user interface [43]. Carbon and oxygen atoms were refined anisotropically, while hydrogen atoms were positioned using SHELX angle-distance constraints and refined in riding approximation. Graphics were prepared with Mercury3.11 [44] and Jmol 14.6.4 [45].

The most important data concerning structure solution and refinement are collected in Table 4.

#### Multi-Temperature X-Ray Data Collection

Single-crystal X-ray diffraction data used to characterize unit-cell changes of 1a with temperature were collected on an Agilent SuperNova single-source diffractometer (Mo radiation, λ = 0.71073 *Å*). A single crystal of 1a was mounted on a glass capillary with epoxy resin. The sample temperature was controlled by an Oxford Cryosystems cooling device. The data were collected, integrated, and corrected for absorption with CrysAlis software (Rigaku Oxford Diffraction, Yarnton, UK) [46]. Unit cell parameters obtained from integrated and corrected data were used. A summary of the obtained unit-cell parameters is presented in Table S1 in the Supporting Information.

### 3.4. High-Pressure X-Ray Data Collection and Refinement

All high-pressure single-crystal X-ray measurements were conducted by using a SuperNova single-source diffractometer (Ag radiation, λ = 0.56085 *Å*). The CrysAlis program (Rigaku Oxford Diffraction, Yarnton, UK) was applied [46] for the data collection and its further reduction. A DAC opening angle cutoff of 32${}^{\circ }$ was used, as the intensities of the few reflections registered beyond that limit were found to be heavily affected by gasket shadowing. The program Absorb7 [47,48] was used for correcting the data for the DAC absorption, gasket shadowing, and absorption of the sample itself. Above 2.0 GPa, the single crystal began to deteriorate, in particular showing increased mosaicity in the b${}^{*}$ direction. This deterioration was irreversible with respect to pressure. As a result, while it was still possible to evaluate unit cell parameters and even tentatively solve the structure, resulting data quality and models were not good enough for publication.

The structures were solved by direct methods using the SHELXS program and refined with SHELXL [42] within the Olex2 graphical environment [43]. Due to the low completeness of the experimental data, the atomic displacement parameters of the carbon atoms in the final structural models had to be restrained to fulfill the Hirshfeld test along the covalent bonds (RIGU instruction in SHELX [49]).

The most important data concerning structure solution and refinement are collected in Table 4. The structural data for 1a at ambient conditions and at non-ambient pressures were deposited with CCDC (1898762, 1898759, 1898761, 1898760 deposition numbers, accordingly).

### 3.5. Spectroscopic Measurements

The UV-Vis luminescence spectra were recorded with a Labram HR800 (Horiba Scientific, Edison, NJ, USA) spectrometer coupled with an Olympus BX61 confocal microscope (Olympus Inc., Shinjuku, Tokyo, Japan) and equipped with a Peltier-cooled CCD detector Synapsis (Horiba Scientific, Edison, NJ, USA), 1024:256 pixel. A diode-pumped, frequency-doubled Nd:YAG laser 532 nm, output laser power: 100 mW (Spectra-Physics, Santa Clara, CA, USA) was utilized as the excitation source. A holographic grating with 600 lines/mm was used. The calibration of the instrument was performed using a 520-cm${}^{-1}$ Raman signal of a silicon wafer. The spectra were collected for the same crystal, which was used for structure determinations, at several pressures. In some cases, the conditions coincided with those for the crystal structure determination. Analogously to the X-ray data, the pressure inside the gasket hole was determined by measuring the reference ruby fluorescence.

### 3.6. Theoretical Calculations

Optimizations of 1a geometries were carried out using periodic ab initio calculations with CRYSTAL09 and CRYSTAL14 software (University of Turin, Torino, Italy) [50]. The B3LYP functional with 6-31G** basis set provided by CRYSTAL14 was used throughout all computations. The level of accuracy in evaluating the Coulomb and exchange series was controlled by five TOLINTEG parameters, for which values of $10^{-7}$, $10^{-7}$, $10^{-7}$, $10^{-9}$, and $10^{-30}$ were used. Radial and angular points of the atomic grid were generated through Gauss–Legendre and Lebedev quadrature schemes. The condition for the Self-Consistent Field (SCF) convergence was set to 10${}^{-8}$ on the total energy difference between two subsequent cycles. The shrinking factor of the reciprocal-space net was set to 8. The total energies obtained with this mesh were fully converged. The crystal symmetry was imposed as a constraint during the whole optimization process.

The following calculations were performed:

(a) geometry optimization using B3LYP-D*, where B3LYP was augmented with an empirical dispersion term as proposed by Grimme [51] and modified for molecular crystals by Civalleri et al. [52]. A full relaxation of both lattice parameters and atomic coordinates by means of analytical energy gradients was applied,
(b) Equations Of State (EOS) calculations were performed for the pressure range from atmospheric up to 4.5 GPa (8 points in total), with full relaxation of both lattice parameters and atomic coordinates by means of analytical energy gradients. The bulk modulus of the solid 1a has been estimated using a third order Birch–Murnaghan-type equation of state [32],
(c) crystal cohesive energy and intermolecular interaction energies were estimated, using the procedure described in the Supporting Information,
(d) Crystalline band structure and partial electronic density of states were calculated with CRYSTAL14 in order to estimate the band gap changes and atomic contributions to frontier orbitals.

### 3.7. Energy Frameworks

Intermolecular interaction energies were calculated between the crystallographically-independent molecule of 1a and all its nearest neighbors in the crystal lattice using CrystalExplorer17 (University of Western Australia, Perth, Australia) [28]. The model (termed CE-B3LYP) used B3LYP/6-31G** wave functions calculated applying molecular geometry extracted from the optimized crystal structure at ambient pressure. Pairwise energies are depicted via energy frameworks, whereby cylinders with thickness proportional to the magnitude of the interaction energy link the center of mass of the molecules. The idea of using energy frameworks to explain and rationalize the mechanical behavior of crystals at the molecular level was discussed by Turner and coworkers [53].

## 4. Conclusions

We investigated the influence of the increased pressure on the monocrystalline sample of a model monosubstituted PAH, 1a. We performed X-ray structural analysis at several pressures in the range of 0.001–2.6 GPa, as well as UV-Vis fluorescence analysis and theoretical calculations. The material turned out to be an indirect semiconductor, with the crystal structure stabilized predominantly by dispersive interactions between molecules organized in infinite π-stacks. π-stacking can be related to the unique spectroscopic properties of the material, namely its increased fluorescence efficiency and red shift of the fluorescence in the solid state. In particular, it appears to facilitate the formation of the efficiently red-emitting multi-component excimers of 1a. It must be stressed, however, that more definite statements concerning the electronic excitations in crystalline 1a could only be made based on a pressure-dependent time-resolved luminescence study, which would allow comparing luminescence decay-times of the orange and red bands.

The calculations confirmed that the band gap of the material depends on the distance between the conjugated π-systems of the flat pyrene moieties and that decreasing this distance straightforwardly decreases the band gap.

It is apparent that a more interesting processes can occur in this material at pressures above 5 GPa, where the interactions between the π-stacked molecules become decidedly repulsive. Verifying what happens with the material in such pressure regimes will be the object of a further study.

## Figures and Tables

**Table 1:** The energies of all intermolecular interactions in the crystal structure of 1a as estimated with CrystalExplorer17 [28] in kJ/mol. The total interaction energies for π-stacked molecules in bold, for the strongest H-bond—underlined.

Symmetry Operation (Translation) ${}^{1}$	E${}_{\mathrm{ele}}$	E${}_{\mathrm{pol}}$	E${}_{\mathrm{dis}}$	E${}_{\mathrm{rep}}$	E${}_{\mathrm{tot}}$${}^{2}$
**−x, −y, −z (0, 0, 0)**	**−13.4**	**−3**	**−101.2**	**68**	**−61.4**
−x, y+1/2, −z+1/2 (0, −1, −1)	0.5	−0.5	−6.5	5.1	−2.3
−x, −y, −z (0, 2, −1)	1.4	−0.2	−4.7	0.6	−2.3
x, −y+1/2, z+1/2 (0, 1, −1)	−6.5	−2.6	−17.4	13.3	−15.6
−x, y+1/2, −z+1/2 (0, −1, 0)	−2.7	−0.6	−8.6	4.7	−7.8
**−x, −y, −z (1, 0, 0)**	**−13.5**	**−3.8**	**−107.7**	**74.3**	**−63.8**
x, y, z (0, 0, 1)	−3.2	−0.9	−14.3	9.1	−10.7
−x, −y, −z (1, 2, 1)	−18	−5.8	−24.5	27.6	−27.8
−x, y+1/2, −z+1/2 (−1, −1, 0)	−4.4	−1.2	−10.1	4.3	−11.5
x, −y+1/2, z+1/2 (0, 2, 0)	−0.8	−0.5	−10.6	8.1	−5.3

${}^{1}$ transformation with respect to the independent molecule in the crystallographic asymmetric unit. ${}^{2}$ The columns report the electrostatic, polarization, dispersion and repulsion terms of the intermolecular interaction energies accordingly; E${}_{\mathrm{tot}}$ is the total interaction energy.

**Table 2:** The temperature (A) and pressure-induced strains: based on experimental data (B) and based on theoretical predictions (C). The α and K indicate accordingly the temperature- or pressure induced strain values along the principal axes, a, b and c indicate crystallographic directions.

**(A)**				
		Component along the Crystallographic Axis		
Principal axis, i	$\alpha (MK^{-1})$	**a**	**b**	**c**
3	100(3)	−0.9498	0	0.3130
2	40(1)	0	1	0
1	22(1)	−0.5189	0	−0.8548
(**B**)				
		Component along the Crystallographic Axis		
Principal axis, i	$K(TPa^{-1})$	**a**	**b**	**c**
1	26(18)	0.9996	0	0.0268
2	17(10)	0	−1	0
3	11(4)	0.1353	0	0.9908
(**C**)				
		Component along the Crystallographic Axis		
Principal axis, i	$K(TPa^{-1})$	**a**	**b**	**c**
1	14.4(0.6)	0.9994	0	−0.0349
2	12.2(1.9)	−0.2095	0	−0.9778
3	6.2(2.6)	0	−1	0

**Table 3:** Variation of the cohesive energy and the energy of the π⋯π stacking interaction of all intermolecular interaction in the crystal structure of 1a as estimated with CRYSTAL14 [35] in kJ/mol. The intermolecular interaction energies from periodic DFT and CrystalExplorer [28] estimation (in brackets) were remarkably close at ambient pressure.

p	E${}_{\mathrm{cohesive}}$	E${}_{\pi \cdots \pi }$
GPa		kJ/mol
0.0001	−130.06	−63.97 (*−63.80*)
0.3	−128.62	−61.70
0.8	−124.72	−56.23
1.3	−119.58	−49.82
1.9	−111.97	−41.38
2.7	−101.45	−30.47
3.5	−87.84	−17.50
4.4	−72.40	−3.58

**Table 4:** Experimental details. The triclinic setting (α and γ cell angles diverging from 90${}^{\circ }$) at 2.6 GPa resulted from inferior data quality at this pressure point (broadening of diffraction spots) and does not indicate a phase transition.

	Ambient	0.8 GPa	1.0 GPa	1.3 GPa	2.2 GPa	2.6 GPa
Empirical formula	C20 H12 O	C20 H12 O	C20 H12 O	C20 H12 O	C20 H12 O	C20 H12 O
Formula weight/g	268.3	268.3	268.3	268.3	268.3	268.3
Crystal system	monoclinic	monoclinic	monoclinic	monoclinic	monoclinic	monoclinic
Space group	P21/c	P21/c	P21/c	P21/c	P21/c	P21/c
a (Å)	7.1164 (2)	7.0094 (6)	7.080 (4)	6.8019 (9)	6.664 (4)	6.63 (3)
b (Å)	22.2299 (7)	21.98 (2)	22.37 (16)	21.80 (4)	21.9 (2)	21.16 (9)
c (Å)	8.4575 (3)	8.3822 (5)	8.438 (4)	8.2116 (9)	8.114 (7)	8.03 (3)
α (${}^{\circ }$)	90	90	90	90	90	88.3 (6)
β (${}^{\circ }$)	98.4040 (10)	98.391 (6)	98.33 (5)	98.044 (11)	97.50 (6)	97.7 (3)
γ (${}^{\circ }$)	90	90	90	90	90	87.2 (7)
Volume (Å${}^{3}$)	1323.58 (7)	1277.7 (13)	1322 (9)	1206 (2)	1173 (14)	1115 (8)
Z	4	4	4	4	4	4
$\rho_{calc}$ (mg/mm${}^{3}$)	1.346	1.395	1.348	1.478	1.520	1.599
F (000)	560	560	560	560	560	560
(μ/mm${}^{-1}$)	0.082	0.055	0.053	0.058	0.060	0.063
Max. transmission	1	1	1	1	-	-
Min. transmission	0.9396	0.739	0.387	0.389	-	-
Abs.correction type	Gaussian	Gaussian	Gaussian	multi-scan	-	-
Crystal color	yellow	yellow	yellow	orange	orange	orange
Crystal size (mm)	0.22	0.339	0.339	0.339	0.339	0.339
	0.18	0.275	0.275	0.275	0.275	0.275
	0.07	0.144	0.144	0.144	0.144	0.144
Data completeness (%)	0.997	0.311	0.3226	0.3	0.3169	0.2344
R${}_{int}$	0.0408	0.08	0.0875	0.1022	0.3725	0.7271
R${}_{sigma}$	0.0214	0.0342	0.0479	0.0561	0.5098	2.321
Index ranges h k l	−12:12	−9:9	−8:8	−9:9	−7:7	−7:7
	−39:38	−6:6	−6:7	−6:6	−6:6	−9:9
	−14:14	−11:11	−10:10	−10:10	−9:9	−9:10
Reflections collected	68,366	14,847	8821	11,618	6191	10,501
2θ range for data collection	1.832:38.661	2.071:21.958	2.055:20.52	2.386:22.074	1.998:19.264	2.955:18.475
Temperature (K)	301 (2)	295.0 (8)	263 (40)	294.5 (3)	294.9 (2)	294.8 (2)
X-ray wavelength (Å)	0.71073	0.56087	0.56087	0.56087	0.56087	0.56087
Independent reflections I	7499	910	826	822	-	-
Independent reflections I > 2 σ(I)	4569	496	413	492	-	-
Largest diff. peak/hole/e (Å${}^{-3}$)	0.471	0.074	0.095	0.097	-	-
	−0.284	−0.084	−0.148	−0.094	-	-
Goof	1.018	1.002	1.035	1.091	-	-
Parameters	191	191	191	191	-	-
Data	7499	910	826	822	-	-
Restraints	0	177	303	177	-	-
R1 all data	0.0963	0.0932	0.1173	0.1045	-	-
R1 [I >= 2σ (I)]	0.0596	0.0348	0.0533	0.0496	-	-
wR2 [I >= 2σ (I)]	0.1721	0.0727	0.1277	0.1055	-	-
wR2 all data	0.2065	0.0944	0.1657	0.1268	-	-

## Supplementary Materials

The supplementary materials are available online.

## Abbreviations

The following abbreviations are used in this manuscript:

PAH	Poly-Aromatic Hydrocarbon
DAC	Diamond Anvil Cell
ADP	Atomic Displacement Parameter
