# Supplementary material for: Pressure-Dependent Structural and Luminescence Properties of 1-(Pyren-1-yl)but-2-yn-1-one
Source: Molecules. 2019 Mar 20;24(6):1107. doi: 10.3390/molecules24061107 (PMC6471252; doi:10.3390/molecules24061107)
Supplement: Supplementary file 1 [file molecules-24-01107-s001.pdf]

Pressure-dependent Structural and Luminescence Properties of 1-(pyren-1-yl)but-2-yn-1-one

Anna Makal [1], * , Joanna Krzeszczakowska [1], and Roman Gajda [1],

[1] University of Warsaw, Faculty of Chemistry, Biological and Chemical Research Centre, ul. Zwirki i Wigury 101, 02-096 Warsaw, Poland.
* Correspondence: amakal@chem.uw.edu.pl; Tel.: +48-22-55-26-769 (A.M.)

1. Multi-temperature unit cell determinations

Table S1. Unit cell parameters of **1a** in function of temperature.

T/K	89.97(15)	110.00(10)	130.00(10)	150.00(10)	170.01(10)	169.99(10)
a/Å	6.9924(4)	7.0050(4)	7.0165(4)	7.0264(4)	7.0378(4)	7.0382(4)
b/Å	22.0678(10)	22.0866(11)	22.1057(11)	22.1173(11)	22.1379(12)	22.1378(12)
c/Å	8.3993(2)	8.4073(3)	8.4134(3)	8.4164(3)	8.4233(3)	8.4213(3)
α/°	90	90	90	90	90	90
β/°	97.870(3)	97.922(4)	97.985(4)	98.046(4)	98.107(4)	98.100(4)
γ/°	90	90	90	90	90	90
V/Å^3	1283.86(10)	1288.34(11)	1292.30(11)	1295.07(11)	1299.26(12)	1299.03(12)
R_{int}	0.0237	0.024	0.024	0.0233	0.0246	0.0234
h	9	9	9	9	9	9
	-8	-8	-8	-8	-8	-8
k	28	28	28	28	28	28
	-25	-25	-25	-25	-25	-25
l	11	11	11	11	11	11
	-11	-11	-11	-11	-11	-11
Reflections collected	6758	6779	6804	6824	6863	6867
2Θ range	30.6274	30.5816	30.5374	30.5156	30.4831	30.4824
	1.8456	1.8441	1.8425	1.8415	1.8398	1.8398

T/K	210.00(10)	230.00(10)	250.00(10)	270.00(10)	284(8)
a/Å	7.0637(4)	7.0786(4)	7.0921(4)	7.1065(4)	7.1265(5)
b/Å	22.1679(13)	22.1850(13)	22.2057(13)	22.2208(13)	22.2526(15)
c/Å	8.4344(3)	8.4391(3)	8.4460(3)	8.4518(4)	8.4600(4)
α/°	90	90	90	90	90
β/°	98.206(4)	98.262(4)	98.315(4)	98.361(5)	98.415(5)
γ/°	90	90	90	90	90
V/Å^3	1307.21(12)	1311.52(12)	1316.13(12)	1320.45(13)	1327.17(15)
R_{int}	0.0229	0.0241	0.0228	0.023	0.0237
h	9	9	9	9	9
	-8	-8	-8	-8	-8
k	28	28	28	28	27
	-25	-25	-25	-25	-25
l	11	11	11	11	11
	-11	-11	-11	-11	-11
Reflections collected	6910	6951	7002	7023	7063
2Θ range	30.4202	30.3849	30.5559	30.543	30.5002
	1.8373	1.8359	1.8342	1.8329	1.8303

2. Theoretical Calculations

2.1 Cohesive and Intermolecular Interaction Energies

Since CRYSTAL09 does not allow to include both dispersion and BSSE corrections during one computational process, whenever an interaction analysis was due, calculations were performed in two different variants in order to consider both errors in the final result. The first variant utilised a GRIMME procedure and was used to obtain both the energy without any corrections and the value of dispersion correction. The second variant used GHOSTS command and aimed to estimate Basis Set Superposition Error. A formula for corrected value of energy becomes then:

$$E_{corrected} = E_{without\ corrections} + \Delta E_{dispersion\ correction} + \Delta E_{BSSE}$$

In order to obtain PA cohesive energy, firstly the total bulk energy was obtained by fixing experimental cell parameters and refining only atomic positions. Then the energy of a single, isolated molecule was calculated based on its fixed geometry obtained in the previous step. The cohesive energy was then estimated as a difference between total crystal energy and twice the energy of a single molecule:

$$E_{cohesive} = E_{bulk} - 2\ E_{molecule}$$

In order to evaluate individual interactions within PA stacks stretched in the (100) direction, energy of investigated dimer formed by inversion-related moieties was calculated based on a fixed optimised bulk geometry and compared to twice the energy of a single monomer:

$$E_{interaction\ between\ dimers} = E_{dimer} - 2\ E_{molecule}$$

The interaction between neighboring stacks was estimated slightly differently. The energy of infinite double rod, composed out of periodic one-dimensional lattice of (100)-translation related dimers, was compared to double the energy of a single infinite rod defined in the same way. In this approach the geometry of individual stacks, dimers and monomers was also not optimized and was exported out of optimized bulk structure mentioned in the previous paragraph instead.

$$\mathrm{E}_{\mathrm{interaction\ between\ stacks}} = \mathrm{E}_{\mathrm{double\ rod}} - 2\ \mathrm{E}_{\mathrm{single\ rod}}$$

2.2 Density of States denotes the number of states (in the present case, the number of electronic energy states) per energy interval, available to be occupied. In particular, partial DOS plots illustrate contributions of selected atoms (or moieties) to the electronic states at the given energy levels. These contributions are based on Mulliken population analysis and orbital overlaps. In the present case, they were calculated with CRYSTAL14, using crystalline orbital overlaps.

While such plots do not represent the shape of orbitals, they give a schematic picture of available energy levels in the system, the position and shape of the HOMO – LUMO gap on the energy axis and an insight into the role of selected moieties in the formation of frontier orbitals.

3. Additional Structural Information

Table S2. Selected interatomic distances and interplanar angles in the crystal structures of **1a**, resulting from theoretical calculations and experimental diffraction data.

**p /GPa**	**$d_{C=O}$/Å**		**$d_{C≡C}$/Å**		**$d_{C1—C17}$/Å**		**$d_{C15—C16}$/Å**		**$d_{C1—C2}$/Å**	
	theor.	exp.	*theor.*	exp.	*theor.*	exp.	*theor.*	exp.	*theor.*	exp.
0.0001	*1.240*	1.2170(15)	*1.214*	1.1931(17)	*1.486*	1.4818(14)	*1.428*	1.4249(13)	*1.426*	1.4199(13)
0.3	*1.240*		*1.213*		*1.485*		*1.428*		*1.426*	
0.4		1.239(8)		1.10(4)		1.50(3)		1.33(3)		1.39(4)
0.8	*1.240*	1.242(5)	*1.213*	1.18(2)	*1.484*	1.491(15)	*1.427*	1.35(2)	*1.425*	1.40(2)
1.3	*1.241*	1.233(6)	*1.212*	1.17(3)	*1.482*	1.47(2)	*1.427*	1.35(3)	*1.424*	1.38(3)
1.9	*1.241*		*1.211*		*1.481*		*1.426*		*1.423*	
2.7	*1.241*		*1.211*		*1.479*		*1.425*		*1.422*	
3.5	*1.241*		*1.210*		*1.477*		*1.424*		*1.421*	
4.4	*1.242*		*1.209*		*1.475*		*1.423*		*1.420*	

**p /GPa**	**$d_{pyrene... pyrene}$ /Å**		lateral shift /Å		**$d_{pyrene... pyrene}$ /Å**		lateral shift /Å		**$d_{C–H...O}$/Å**			
									C3 … O1		C20 … O1	
	theor.	exp.	*theor.*	exp.	*theor.*	exp.	*theor.*	exp.	*theor.*	exp.	*theor.*	exp.
0.0001	*3.420*	3.464(3)	*0.81*	0.803(4)	*3.364*	3.401(3)	*1.64*	1.677(4)	*3.340*	3.411(2)	*3.392*	3.531(2)
0.3	*3.390*		*0.80*		*3.330*		*1.62*		*3.286*		*3.365*	
0.4		3.45(5)		0.80(4)		3.38(5)		1.67(4)		3.41(1)		3.56(2)
0.8	*3.357*	3.41(4)	*0.80*	0.79(5)	*3.294*	3.34(4)	*1.61*	1.63(4)	*3.237*	3.361(8)	*3.335*	3.48(1)
1.3	*3.322*	3.24(5)	*0.80*	0.79(5)	*3.256*	3.31(5)	*1.60*	1.63(5)	*3.190*	3.26(1)	*3.306*	3.38(1)
1.9	*3.286*		*0.80*		*3.219*		*1.59*		*3.146*		*3.278*	
2.7	*3.246*		*0.80*		*3.179*		*1.58*		*3.105*		*3.248*	
3.5	*3.205*		*0.80*		*3.140*		*1.57*		*3.064*		*3.222*	
4.4	*3.167*		*0.80*		*3.105*		*1.57*		*3.029*		*3.195*	

**p /GPa**	**pyrene bend /°**		**O1-C17-C1-C2 /°**		**< pyrene pyrene /°**		**inclination to [100]/°**	
	theor.	exp.	*theor.*	exp.	*theor.*	exp.	*theor.*	exp.
0.0001	*4.12*	3.3(2)	*-8.22*	-8.0(2)	*28.31*	28.99(11)	*75.85*	75.5(5)
0.3	*4.12*		*-7.84*		*28.36*		*75.82*	
0.4		3.6(9)		-8(2)		29(1)		75(1)
0.8	*4.16*	3.8(9)	*-7.38*	-7(1)	*28.44*	29.9(9)	*75.78*	75.3(9)
1.3	*4.22*	3.9(9)	*-7.00*	-7(2)	*28.57*	30(1)	*75.72*	75(1)
1.9	*4.23*		*-6.57*		*28.66*		*75.67*	
2.7	*4.32*		*-6.10*		*29*		*75.50*	
3.5	*4.33*		*-5.51*		*29.52*		*75.24*	
4.4	*4.35*		*-5.06*		*29.87*		*75.06*	

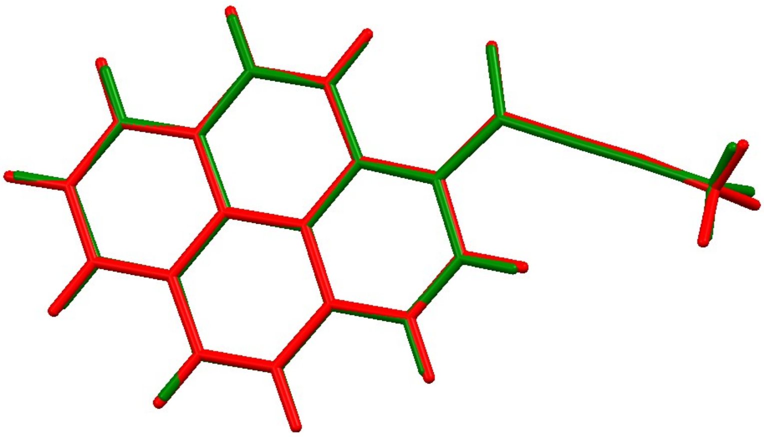

Figure S1. The closest overlay of experimental (green) and theoretically predicted (red) molecular geometries of **1a** at 0.8 GPa. The major differences between the experimental and calculated geometry are the C–H bond lengths and a slight rotation of the terminal methyl group. RMS deviation between these structures is of the order of 0.05Å.

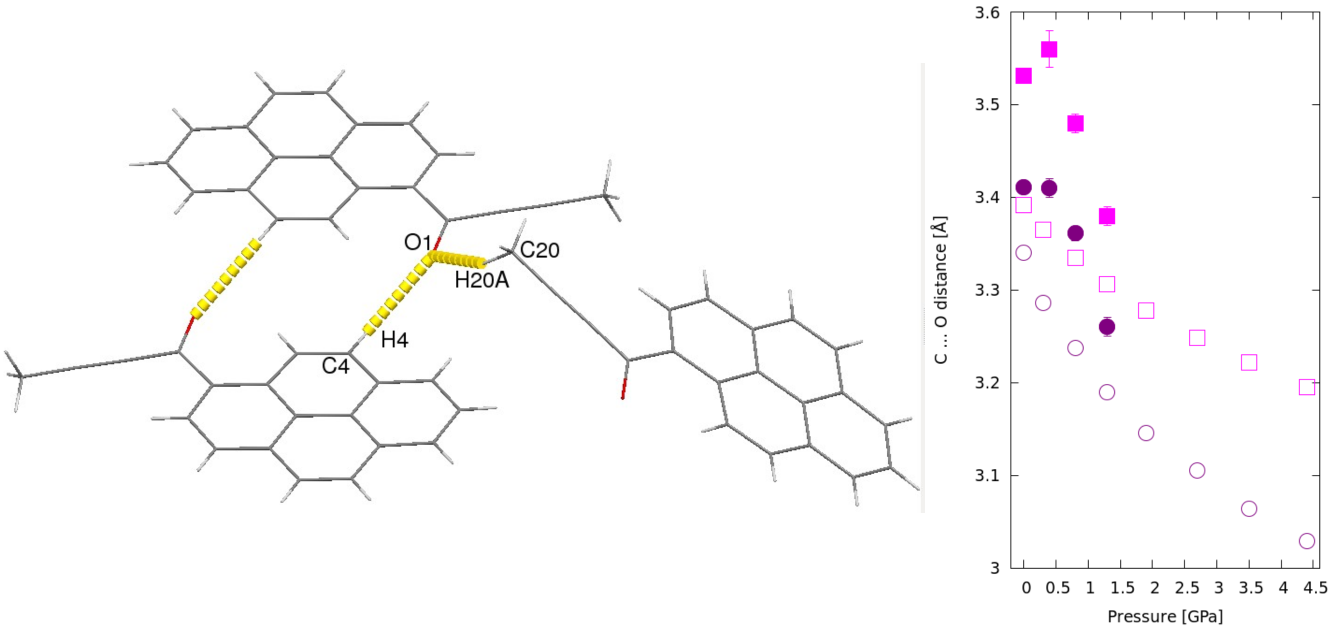

(a) (b)

Figure S2. (a) selected intermolecular H-bonds in **1a**; (b) Variation of the C…O distances in selected intermolecular H-bonds in **1a** with pressure. Experimental results represented by filled markers with uncertainties. The purple dots and magenta squares refer to C…O distances of the C4–H4…O1 and C20–H20A…O1 H-bonds accordingly.

Table S3. The energies of the Highest Occupied Crystalline Orbitals (HOCO) and Lowest Unoccupied Crystalline Orbitals (LUCO), resulting band gap and predicted wavelength for maximal absorption in **1a**.

**1a**	**Isolated molecule**	**p / GPa**							
		0.0001	**0.5**	**0.8**	**1.3**	**2.0**	**2.5**	**3.0**	**4.0**
LUCO* eV	-2.655	-2.599	-2.616	-2.621	-2.625	-2.626	-2.623	-2.617	-2.607
HOCO* eV	-5.942	-5.132	-5.075	-5.049	-5.009	-4.952	-4.915	-4.875	-4.807
Gap / eV	**3.287**	**2.534**	**2.459**	**2.428**	**2.384**	**2.326**	**2.292**	**2.258**	**2.200**
Gap / hartree		0.093	0.090	0.089	0.088	0.085	0.084	0.083	0.081
λ_{max}^{abs} / nm	**377**	**489**	**504**	**511**	**520**	**533**	**541**	**549**	**564**

*HOMO / LUMO in the case of isolated molecule

4. Structural models obtained from periodic DFT calculations at several pressures.

Table S4. Coordinates of atoms in **1a** structures optimized at various pressures by CRYSTAL09.

```
data_1a_0.0001GPa
_symmetry_cell_setting           monoclinic
_symmetry_space_group_name_H-M   'P 21/c'
_symmetry_Int_Tables_number      14
loop_
_symmetry_equiv_pos_site_id
_symmetry_equiv_pos_as_xyz
1 x,y,z
2 -x,1/2+y,1/2-z
3 -x,-y,-z
4 x,1/2-y,1/2+z
_cell_length_a                   7.02897529
_cell_length_b                   21.99879741
_cell_length_c                   8.36002453
_cell_angle_alpha                90
_cell_angle_beta                 98.024772
_cell_angle_gamma                90
_cell_volume                     1280.043869
loop_
_atom_site_label
_atom_site_type_symbol
_atom_site_fract_x
_atom_site_fract_y
_atom_site_fract_z
O(1)    O  3.065681874592E-01 -1.250946245705E-01  3.774850686824E-01
C(1)    C  2.222579070915E-01 -8.255469341091E-02  1.098998710751E-01
C(2)    C  2.722938099703E-01 -2.069399319677E-02  1.452492937029E-01
C(3)    C  3.267700579333E-01  2.473772531872E-03  3.057731888901E-01
C(4)    C  3.807251637410E-01  6.153177279370E-02  3.331105796470E-01
C(5)    C  3.844875043556E-01  1.037330057820E-01  2.035036627427E-01
C(6)    C  4.505961507530E-01  1.637719057790E-01  2.296379312736E-01
C(7)    C  4.501852262383E-01  2.038037310867E-01  1.004249782070E-01
C(8)    C  3.815570712822E-01  1.847745108715E-01 -5.610853349695E-02
C(9)    C  3.187994575771E-01  1.249025303020E-01 -8.749883687537E-02
C(10)   C  2.549223034659E-01  1.039051763147E-01 -2.484026839495E-01
C(11)   C  2.001983953568E-01  4.520171501378E-02 -2.772126832788E-01
C(12)   C  2.060364061414E-01  2.075749272289E-03 -1.480395820332E-01
C(13)   C  1.551136237401E-01 -5.885580641834E-02 -1.775990428563E-01
C(14)   C  1.633039904766E-01 -9.979192438131E-02 -5.141081918587E-02
C(15)   C  2.661081267295E-01  2.136019667190E-02  1.409021009724E-02
C(16)   C  3.217536659606E-01  8.325534877579E-02  4.340781521643E-02
C(17)   C  2.338253705959E-01 -1.311139093491E-01  2.344522810009E-01
C(18)   C  1.555102612120E-01 -1.899017006966E-01  1.824052531107E-01
C(19)   C  8.845336833074E-02 -2.394122926515E-01  1.432660767752E-01
C(20)   C  4.062506349219E-03 -2.969738155970E-01  8.513906793054E-02
H(3)    H  3.269200990407E-01 -2.844443098846E-02  4.058964414042E-01
H(4)    H  4.266666423195E-01  7.726769177283E-02  4.552522624941E-01
H(6)    H -4.940727891593E-01  1.778081572255E-01  3.518446617797E-01
H(7)    H -4.931504471002E-01  2.495022861968E-01  1.209733522380E-01
H(8)    H  3.781603144635E-01  2.162473875449E-01 -1.561855264369E-01
H(10)   H  2.541913863159E-01  1.357138335078E-01 -3.474992115053E-01
H(11)   H  1.530015057827E-01  2.967482211112E-02 -3.995080924801E-01
H(13)   H  1.108248519174E-01 -7.369916452996E-02 -3.008664430096E-01
H(14)   H  1.270487769645E-01 -1.469504758480E-01 -7.667550776913E-02
H(20A)  H  1.142106403637E-01 -3.269411571281E-01  4.748035263015E-02
H(20B)  H -1.032659950154E-01 -2.888516007058E-01 -2.068609423129E-02
H(20C)  H -6.675863691139E-02 -3.198701204311E-01  1.769975535120E-01
```

```
data_1a_0.3GPa
_symmetry_cell_setting           monoclinic
_symmetry_space_group_name_H-M   'P 21/c'
_symmetry_Int_Tables_number      14
loop_
_symmetry_equiv_pos_site_id
_symmetry_equiv_pos_as_xyz
1 x,y,z
2 -x,1/2+y,1/2-z
3 -x,-y,-z
4 x,1/2-y,1/2+z
_cell_length_a                   6.96299120
_cell_length_b                   21.93036063
_cell_length_c                   8.27589613
_cell_angle_alpha                90
_cell_angle_beta                 98.207091
_cell_angle_gamma                90
_cell_volume                     1250.794384
loop_
_atom_site_label
_atom_site_type_symbol
_atom_site_fract_x
_atom_site_fract_y
_atom_site_fract_z
O(1)    O  3.062288552725E-01 -1.247695749811E-01  3.801628298705E-01
C(1)    C  2.213186769977E-01 -8.280419849333E-02  1.088087258325E-01
C(2)    C  2.718038369814E-01 -2.070432330552E-02  1.436163147200E-01
C(3)    C  3.274139961294E-01  2.839321545558E-03  3.054047398016E-01
```

```
C(4)   C  3.817110092726E-01  6.213019680952E-02  3.321831645404E-01
C(5)   C  3.847601622125E-01  1.042014864254E-01  2.007142377218E-01
C(6)   C  4.514683213141E-01  1.644680532570E-01  2.263095683399E-01
C(7)   C  4.505099877006E-01  2.043462331878E-01  9.517575820144E-02
C(8)   C  3.807037327034E-01  1.849292823394E-01 -6.269053157612E-02
C(9)   C  3.174799573326E-01  1.248215935235E-01 -9.352434591247E-02
C(10)  C  2.526328714963E-01  1.034104146097E-01 -2.557587237842E-01
C(11)  C  1.971586030320E-01  4.450758673194E-02 -2.840284350250E-01
C(12)  C  2.037332312492E-01  1.523186269604E-03 -1.529330883164E-01
C(13)  C  1.524627430328E-01 -5.963874970340E-02 -1.820085551295E-01
C(14)  C  1.611990333431E-01 -1.004389126194E-01 -5.391813517070E-02
C(15)  C  2.649429950457E-01  2.120065973715E-02  1.055003342190E-02
C(16)  C  3.209489679537E-01  8.333572927168E-02  3.929509321266E-02
C(17)  C  2.333701093781E-01 -1.311807341235E-01  2.354242128596E-01
C(18)  C  1.552910096462E-01 -1.902882736033E-01  1.834645638268E-01
C(19)  C  8.718863050315E-02 -2.399085788576E-01  1.440104416517E-01
C(20)  C  8.816263987674E-04 -2.973695645301E-01  8.448701612584E-02
H(3)   H  3.280964519634E-01 -2.797353815883E-02  4.069824383319E-01
H(4)   H  4.288301931511E-01  7.814263120991E-02  4.552697797413E-01
H(6)   H -4.920390047129E-01  1.787319525754E-01  3.495841809518E-01
H(7)   H -4.921707977433E-01  2.501997547614E-01  1.152417356638E-01
H(8)   H  3.768047773587E-01  2.162836285754E-01 -1.642478095230E-01
H(10)  H  2.518910587755E-01  1.350513146667E-01 -3.563492640817E-01
H(11)  H  1.491970910533E-01  2.869723758911E-02 -4.073712205476E-01
H(13)  H  1.075701198368E-01 -7.471772092714E-02 -3.064201034016E-01
H(14)  H  1.245302772679E-01 -1.477828544106E-01 -7.869150046182E-02
H(20A) H  1.120423929978E-01 -3.280366439806E-01  4.934572059696E-02
H(20B) H -1.042536125481E-01 -2.888737751439E-01 -2.453957748398E-02
H(20C) H -7.460390921226E-02 -3.197104496464E-01  1.755553670566E-01

data_1a_0.8GPa
_symmetry_cell_setting           monoclinic
_symmetry_space_group_name_H-M   'P 21/c'
_symmetry_Int_Tables_number      14
loop_
_symmetry_equiv_pos_site_id
_symmetry_equiv_pos_as_xyz
1 x,y,z
2 -x,1/2+y,1/2-z
3 -x,-y,-z
4 x,1/2-y,1/2+z
_cell_length_a                   6.89312824
_cell_length_b                   21.85002635
_cell_length_c                   8.19776680
_cell_angle_alpha                90
_cell_angle_beta                 98.255676
_cell_angle_gamma                90
_cell_volume                     1221.911859
loop_
_atom_site_label
_atom_site_type_symbol
_atom_site_fract_x
_atom_site_fract_y
_atom_site_fract_z
O(1)   O  3.050807901343E-01 -1.244736732205E-01  3.829915499936E-01
C(1)   C  2.203608149048E-01 -8.303587397427E-02  1.081602783591E-01
C(2)   C  2.713748295294E-01 -2.068610485739E-02  1.424623280007E-01
C(3)   C  3.279616132044E-01  3.185553271771E-03  3.053705382807E-01
C(4)   C  3.827225703521E-01  6.271626273129E-02  3.316297071406E-01
C(5)   C  3.853415999328E-01  1.047124145060E-01  1.985009587321E-01
C(6)   C  4.528691588703E-01  1.652056451901E-01  2.235917548120E-01
C(7)   C  4.515875611154E-01  2.049891509220E-01  9.072220584204E-02
C(8)   C  3.805818131159E-01  1.852438900220E-01 -6.830892149510E-02
C(9)   C  3.167385486262E-01  1.248938246183E-01 -9.861104734143E-02
C(10)  C  2.510481268383E-01  1.031184046983E-01 -2.620200964231E-01
C(11)  C  1.948203518972E-01  4.399870910962E-02 -2.897888743973E-01
C(12)  C  2.019223757667E-01  1.094291263422E-03 -1.569853439329E-01
C(13)  C  1.502942546593E-01 -6.031459241247E-02 -1.856350529560E-01
C(14)  C  1.592840755687E-01 -1.010321557013E-01 -5.582807716782E-02
C(15)  C  2.640851962827E-01  2.112214755192E-02  7.691992306293E-03
C(16)  C  3.205140551883E-01  8.351049208026E-02  3.592141446020E-02
C(17)  C  2.325496241637E-01 -1.312651024984E-01  2.366575945358E-01
C(18)  C  1.548101154158E-01 -1.907083626447E-01  1.848325542851E-01
C(19)  C  8.559760567857E-02 -2.404626683335E-01  1.452580747339E-01
C(20)  C -2.760439159019E-03 -2.978408644572E-01  8.462706837586E-02
H(3)   H  3.289583296436E-01 -2.757108649611E-02  4.082354754167E-01
H(4)   H  4.308562232780E-01  7.896918324470E-02  4.555495183906E-01
H(6)   H -4.894674765366E-01  1.796434083694E-01  3.478034065608E-01
H(7)   H -4.902374012461E-01  2.509946609611E-01  1.102948256366E-01
H(8)   H  3.763541819871E-01  2.165228327053E-01 -1.712068701331E-01
H(10)  H  2.503595362294E-01  1.346488865106E-01 -3.639362931507E-01
H(11)  H  1.462335700037E-01  2.793881053539E-02 -4.140594145022E-01
H(13)  H  1.050201633622E-01 -7.560551929587E-02 -3.110787289900E-01
H(14)  H  1.221571660885E-01 -1.485753674206E-01 -8.013374725494E-02
H(20A) H  1.090848973305E-01 -3.290343914233E-01  5.046486199094E-02
H(20B) H -1.069634268243E-01 -2.889059094576E-01 -2.642427358547E-02
H(20C) H -8.145958887752E-02 -3.198509173042E-01  1.755120913578E-01

data_1a_1.3GPa
_symmetry_cell_setting           monoclinic
_symmetry_space_group_name_H-M   'P 21/c'
```

```
_symmetry_Int_Tables_number      14
loop_
_symmetry_equiv_pos_site_id
_symmetry_equiv_pos_as_xyz
1 x,y,z
2 -x,1/2+y,1/2-z
3 -x,-y,-z
4 x,1/2-y,1/2+z
_cell_length_a                   6.82078850
_cell_length_b                   21.75824710
_cell_length_c                   8.12211158
_cell_angle_alpha                90
_cell_angle_beta                 98.174620
_cell_angle_gamma                90
_cell_volume                     1193.142011
loop_
_atom_site_label
_atom_site_type_symbol
_atom_site_fract_x
_atom_site_fract_y
_atom_site_fract_z
O(1)   O  3.042233029260E-01 -1.243240186380E-01  3.858592764861E-01
C(1)   C  2.194744481043E-01 -8.333220645634E-02  1.078596802379E-01
C(2)   C  2.710938534439E-01 -2.071540057699E-02  1.416381432661E-01
C(3)   C  3.286434703883E-01  3.454225895307E-03  3.055589094242E-01
C(4)   C  3.839432878375E-01  6.324537980868E-02  3.312883098938E-01
C(5)   C  3.862557823422E-01  1.052101221401E-01  1.965934125009E-01
C(6)   C  4.548001938490E-01  1.659377475163E-01  2.211311587839E-01
C(7)   C  4.532274375649E-01  2.056713045128E-01  8.661306895040E-02
C(8)   C  3.808634573750E-01  1.856360816980E-01 -7.342022156564E-02
C(9)   C  3.162699870526E-01  1.250349584759E-01 -1.031481136361E-01
C(10)  C  2.496510206837E-01  1.029329509742E-01 -2.675939440890E-01
C(11)  C  1.925588066816E-01  4.358297115129E-02 -2.948491780257E-01
C(12)  C  2.001772731325E-01  7.038815810129E-04 -1.604550268213E-01
C(13)  C  1.481092105491E-01 -6.096959434732E-02 -1.886676567231E-01
C(14)  C  1.573340787511E-01 -1.016587505927E-01 -5.725109135451E-02
C(15)  C  2.633972650234E-01  2.104642931872E-02  5.285560467559E-03
C(16)  C  3.203208504489E-01  8.370563308143E-02  3.298911292653E-02
C(17)  C  2.317995284636E-01 -1.314534222267E-01  2.381294044727E-01
C(18)  C  1.541511908517E-01 -1.912193970827E-01  1.865869217382E-01
C(19)  C  8.367576292278E-02 -2.411057087371E-01  1.469550033806E-01
C(20)  C -6.835235214793E-03 -2.983955650911E-01  8.531615981164E-02
H(3)   H  3.298657032860E-01 -2.728578453381E-02  4.096316936911E-01
H(4)   H  4.329962471526E-01  7.972562046682E-02  4.559538233499E-01
H(6)   H -4.862395819229E-01  1.805067343399E-01  3.461593233134E-01
H(7)   H -4.875506188625E-01  2.518275466878E-01  1.055973542010E-01
H(8)   H  3.763129641894E-01  2.168749473757E-01 -1.775779372960E-01
H(10)  H  2.490617437300E-01  1.343942637452E-01 -3.707332873833E-01
H(11)  H  1.432353448982E-01  2.730106340000E-02 -4.199478497253E-01
H(13)  H  1.023606577794E-01 -7.644822566030E-02 -3.150474523894E-01
H(14)  H  1.196704660183E-01 -1.494153203716E-01 -8.108621978302E-02
H(20A) H  1.055679997982E-01 -3.300195742571E-01  5.123228454702E-02
H(20B) H -1.107166231921E-01 -2.889538201156E-01 -2.706665268033E-02
H(20C) H -8.806178920857E-02 -3.202196429282E-01  1.763548016445E-01

data_1a_1.9GPa
_symmetry_cell_setting           monoclinic
_symmetry_space_group_name_H-M   'P 21/c'
_symmetry_Int_Tables_number      14
loop_
_symmetry_equiv_pos_site_id
_symmetry_equiv_pos_as_xyz
1 x,y,z
2 -x,1/2+y,1/2-z
3 -x,-y,-z
4 x,1/2-y,1/2+z
_cell_length_a                   6.74947104
_cell_length_b                   21.67031753
_cell_length_c                   8.04691040
_cell_angle_alpha                90
_cell_angle_beta                 97.993794
_cell_angle_gamma                90
_cell_volume                     1165.530284
loop_
_atom_site_label
_atom_site_type_symbol
_atom_site_fract_x
_atom_site_fract_y
_atom_site_fract_z
O(1)   O  3.036526949260E-01 -1.241725962273E-01  3.887739077349E-01
C(1)   C  2.187855112861E-01 -8.364035637262E-02  1.076943728018E-01
C(2)   C  2.710357567275E-01 -2.077281406241E-02  1.408334310730E-01
C(3)   C  3.298095618290E-01  3.677957388317E-03  3.056505103388E-01
C(4)   C  3.855371739605E-01  6.372518883003E-02  3.307284528338E-01
C(5)   C  3.872498731240E-01  1.056629753481E-01  1.944542365122E-01
C(6)   C  4.566489748123E-01  1.666285132278E-01  2.182961143950E-01
C(7)   C  4.545957320241E-01  2.063051523552E-01  8.212007301193E-02
C(8)   C  3.808157282295E-01  1.859735828551E-01 -7.879037663137E-02
C(9)   C  3.155908432056E-01  1.251285298780E-01 -1.077996730670E-01
C(10)  C  2.479920050942E-01  1.026954964960E-01 -2.731552949995E-01
C(11)  C  1.900357861604E-01  4.312989677356E-02 -2.997621686394E-01
C(12)  C  1.982665559703E-01  2.899077111275E-04 -1.637645069832E-01
```

```
C(13)  C  1.457444124506E-01 -6.163201424149E-02 -1.914333362271E-01
C(14)  C  1.553681370722E-01 -1.022882784426E-01 -5.841058294172E-02
C(15)  C  2.627251127747E-01  2.094143152652E-02  2.906157235193E-03
C(16)  C  3.200876582597E-01  8.386066105805E-02  2.995340089806E-02
C(17)  C  2.313563960954E-01 -1.316421138336E-01  2.397357765044E-01
C(18)  C  1.539113204274E-01 -1.917201101887E-01  1.885997512758E-01
C(19)  C  8.208017139405E-02 -2.417078401250E-01  1.489554596208E-01
C(20)  C -1.054609863237E-02 -2.988803929686E-01  8.636179788907E-02
H(3)   H  3.314778193966E-01 -2.705329111275E-02  4.109402400135E-01
H(4)   H  4.356169856693E-01  8.043649506174E-02  4.560381657037E-01
H(6)   H -4.831145534511E-01  1.813359578108E-01  3.440427486345E-01
H(7)   H -4.852505827118E-01  2.526146789221E-01  1.003939131942E-01
H(8)   H  3.759032501428E-01  2.171650807398E-01 -1.842047857840E-01
H(10)  H  2.474865504766E-01  1.340755478442E-01 -3.775280779731E-01
H(11)  H  1.399102594444E-01  2.662043734650E-02 -4.255950166505E-01
H(13)  H  9.933335027903E-02 -7.728842213501E-02 -3.186596194117E-01
H(14)  H  1.171130035080E-01 -1.502439285332E-01 -8.167087104469E-02
H(20A) H  1.024571580266E-01 -3.308772168374E-01  5.208884855938E-02
H(20B) H -1.142370025997E-01 -2.889076915319E-01 -2.712480110585E-02
H(20C) H -9.413516669995E-02 -3.205693383851E-01  1.777521175078E-01
```

data_**1a_2.7GPa**

```
_symmetry_cell_setting           monoclinic
_symmetry_space_group_name_H-M   'P 21/c'
_symmetry_Int_Tables_number      14
loop_
_symmetry_equiv_pos_site_id
_symmetry_equiv_pos_as_xyz
1 x,y,z
2 -x,1/2+y,1/2-z
3 -x,-y,-z
4 x,1/2-y,1/2+z
_cell_length_a                   6.67564913
_cell_length_b                   21.55750527
_cell_length_c                   7.97587332
_cell_angle_alpha                90
_cell_angle_beta                 97.773299
_cell_angle_gamma                90
_cell_volume                     1137.263395
loop_
_atom_site_label
_atom_site_type_symbol
_atom_site_fract_x
_atom_site_fract_y
_atom_site_fract_z
O(1)   O  3.018082377220E-01 -1.241299737105E-01  3.917776397943E-01
C(1)   C  2.175787240709E-01 -8.401195560822E-02  1.077478723207E-01
C(2)   C  2.708237845456E-01 -2.085610387616E-02  1.403011021327E-01
C(3)   C  3.308137265189E-01  3.838423887828E-03  3.059500660105E-01
C(4)   C  3.874807820696E-01  6.416055663955E-02  3.304716816339E-01
C(5)   C  3.891175162733E-01  1.061337523772E-01  1.927880911967E-01
C(6)   C  4.600993152675E-01  1.673296353421E-01  2.160012999551E-01
C(7)   C  4.578888697823E-01  2.070232878955E-01  7.830999945844E-02
C(8)   C  3.824263564599E-01  1.864538532562E-01 -8.337415896790E-02
C(9)   C  3.160560086593E-01  1.253491705648E-01 -1.117409289597E-01
C(10)  C  2.474084884583E-01  1.026330752272E-01 -2.779434453931E-01
C(11)  C  1.881933478726E-01  4.282694945787E-02 -3.040008793176E-01
C(12)  C  1.966388573147E-01 -5.181043516862E-05 -1.665778013055E-01
C(13)  C  1.433307198726E-01 -6.225953631927E-02 -1.937774137624E-01
C(14)  C  1.529463283778E-01 -1.029565526734E-01 -5.928037194028E-02
C(15)  C  2.623329151020E-01  2.087158628269E-02  9.622982392109E-04
C(16)  C  3.205866425615E-01  8.407861936743E-02  2.747085627900E-02
C(17)  C  2.299127173276E-01 -1.319296160267E-01  2.414619432149E-01
C(18)  C  1.523828497588E-01 -1.923456180927E-01  1.907781546603E-01
C(19)  C  7.886038192204E-02 -2.424451543494E-01  1.510609809783E-01
C(20)  C -1.626851838052E-02 -2.994550084437E-01  8.719637229238E-02
H(3)   H  3.325265949160E-01 -2.692920467584E-02  4.123204465155E-01
H(4)   H  4.386899521030E-01  8.103153352095E-02  4.563883955315E-01
H(6)   H -4.780496644231E-01  1.821013835798E-01  3.423805582451E-01
H(7)   H -4.804089353518E-01  2.534729388201E-01  9.580499351036E-02
H(8)   H  3.772932706840E-01  2.176566348058E-01 -1.899425022401E-01
H(10)  H  2.473193036810E-01  1.339833255510E-01 -3.834166826557E-01
H(11)  H  1.371957442233E-01  2.614303611677E-02 -4.305199165855E-01
H(13)  H  9.634705896679E-02 -7.808183764992E-02 -3.217965092169E-01
H(14)  H  1.138724053504E-01 -1.511474624789E-01 -8.204982918639E-02
H(20A) H  9.690614692498E-02 -3.316085754649E-01  5.055198501850E-02
H(20B) H -1.213383427920E-01 -2.886427163037E-01 -2.607286245936E-02
H(20C) H -1.006594096982E-01 -3.214082253800E-01  1.793249818234E-01
```

data_**1a_3.5GPa**

```
_symmetry_cell_setting           monoclinic
_symmetry_space_group_name_H-M   'P 21/c'
_symmetry_Int_Tables_number      14
loop_
_symmetry_equiv_pos_site_id
_symmetry_equiv_pos_as_xyz
1 x,y,z
2 -x,1/2+y,1/2-z
3 -x,-y,-z
4 x,1/2-y,1/2+z
_cell_length_a                   6.60776297
```

```
_cell_length_b                    21.44374597
_cell_length_c                    7.89755812
_cell_angle_alpha                 90
_cell_angle_beta                  97.451668
_cell_angle_gamma                 90
_cell_volume                      1109.595228
loop_
_atom_site_label
_atom_site_type_symbol
_atom_site_fract_x
_atom_site_fract_y
_atom_site_fract_z
O(1)    O  2.997427031238E-01 -1.239338309651E-01  3.951544322180E-01
C(1)    C  2.160713651701E-01 -8.437101572588E-02  1.078017019305E-01
C(2)    C  2.707743049857E-01 -2.093915815687E-02  1.393272496280E-01
C(3)    C  3.329114172520E-01  4.049285559975E-03  3.056467781714E-01
C(4)    C  3.906617078625E-01  6.464543055408E-02  3.291724447336E-01
C(5)    C  3.916292203068E-01  1.065904807597E-01  1.897588762441E-01
C(6)    C  4.642433323537E-01  1.680188269433E-01  2.118954873206E-01
C(7)    C  4.615972426390E-01  2.076577386706E-01  7.238834493440E-02
C(8)    C  3.841220937806E-01  1.867739384352E-01 -8.995244605310E-02
C(9)    C  3.164595234765E-01  1.254173249742E-01 -1.172339294796E-01
C(10)   C  2.464045399357E-01  1.023315990983E-01 -2.841612085583E-01
C(11)   C  1.856691280730E-01  4.230131359319E-02 -3.092090910906E-01
C(12)   C  1.943448296975E-01 -5.321977092402E-04 -1.700253208781E-01
C(13)   C  1.398251465993E-01 -6.300346009309E-02 -1.963441380431E-01
C(14)   C  1.495870723622E-01 -1.036678698673E-01 -6.004936060806E-02
C(15)   C  2.617946583849E-01  2.073257598590E-02 -1.742132534447E-03
C(16)   C  3.212203136936E-01  8.421699221680E-02  2.376167971758E-02
C(17)   C  2.281934598196E-01 -1.321235007843E-01  2.435596223928E-01
C(18)   C  1.503495902055E-01 -1.928674168222E-01  1.936997302149E-01
C(19)   C  7.518533788222E-02 -2.430471647592E-01  1.536927053566E-01
C(20)   C -2.259256929310E-02 -2.998002537968E-01  8.826644192495E-02
H(3)    H  3.354509915113E-01 -2.671840397860E-02  4.133503999971E-01
H(4)    H  4.436627444543E-01  8.173697926587E-02  4.555239079602E-01
H(6)    H -4.720247365118E-01  1.829043501839E-01  3.388032808386E-01
H(7)    H -4.751324784185E-01  2.542427386320E-01  8.880908598720E-02
H(8)    H  3.787812138184E-01  2.178961764985E-01 -1.979665586702E-01
H(10)   H  2.464237198336E-01  1.335668937451E-01 -3.910544669566E-01
H(11)   H  1.336682225199E-01  2.539336180225E-02 -4.363581536577E-01
H(13)   H  9.179195043743E-02 -7.902857352649E-02 -3.250892621776E-01
H(14)   H  1.092252183479E-01 -1.520737083011E-01 -8.198570015887E-02
H(20A)  H  9.072626558305E-02 -3.322368880089E-01  4.991692807079E-02
H(20B)  H -1.279663607995E-01 -2.880577440422E-01 -2.521260364021E-02
H(20C)  H -1.087424975965E-01 -3.219066701213E-01  1.808797046846E-01
```

data_**1a_4.4GPa**

```
_symmetry_cell_setting            monoclinic
_symmetry_space_group_name_H-M    'P 21/c'
_symmetry_Int_Tables_number       14
loop_
_symmetry_equiv_pos_site_id
_symmetry_equiv_pos_as_xyz
1 x,y,z
2 -x,1/2+y,1/2-z
3 -x,-y,-z
4 x,1/2-y,1/2+z
_cell_length_a                    6.54274636
_cell_length_b                    21.3437497
_cell_length_c                    7.82686000
_cell_angle_alpha                 90
_cell_angle_beta                  97.149644
_cell_angle_gamma                 90
_cell_volume                      1084.49687
loop_
_atom_site_label
_atom_site_type_symbol
_atom_site_fract_x
_atom_site_fract_y
_atom_site_fract_z
O(1)    O  2.982574640464E-01 -1.239903753305E-01  3.977601642521E-01
C(1)    C  2.148410510843E-01 -8.472982485000E-02  1.076568153992E-01
C(2)    C  2.706503707618E-01 -2.107112629830E-02  1.386473673185E-01
C(3)    C  3.342310742219E-01  4.061446500259E-03  3.057706683265E-01
C(4)    C  3.929784191885E-01  6.488063526084E-02  3.287774612775E-01
C(5)    C  3.937614400311E-01  1.068856485740E-01  1.880497272439E-01
C(6)    C  4.677852221702E-01  1.685112522869E-01  2.096386953975E-01
C(7)    C  4.648124167510E-01  2.082115168871E-01  6.875018828298E-02
C(8)    C  3.856709682961E-01  1.871664057939E-01 -9.432365051489E-02
C(9)    C  3.170211760611E-01  1.255940194596E-01 -1.210380706788E-01
C(10)   C  2.459948991297E-01  1.022952114816E-01 -2.887801946091E-01
C(11)   C  1.838801118014E-01  4.208524257210E-02 -3.133151908081E-01
C(12)   C  1.925900680355E-01 -8.142637014107E-04 -1.728226895576E-01
C(13)   C  1.372038689824E-01 -6.351773860921E-02 -1.987486088963E-01
C(14)   C  1.470092763465E-01 -1.042492402716E-01 -6.105328537933E-02
C(15)   C  2.614389852788E-01  2.063847352586E-02 -3.726650217805E-03
C(16)   C  3.218487516308E-01  8.435157126277E-02  2.129464639263E-02
C(17)   C  2.268777847808E-01 -1.324214274882E-01  2.449703131503E-01
C(18)   C  1.489469207210E-01 -1.934376454472E-01  1.955154027440E-01
C(19)   C  7.199839844960E-02 -2.436857061509E-01  1.556887863356E-01
C(20)   C -2.783567825135E-02 -3.002785397267E-01  8.920233839563E-02
H(3)    H  3.367063245841E-01 -2.674930544116E-02  4.145198156482E-01
```

H(4)	H	4.471341689232E-01	8.208814715464E-02	4.556786434736E-01
H(6)	H	-4.669222676374E-01	1.833946817389E-01	3.371470530692E-01
H(7)	H	-4.706353504076E-01	2.549229119854E-01	8.451112537813E-02
H(8)	H	3.800517595633E-01	2.183239007402E-01	-2.034146763928E-01
H(10)	H	2.468264100449E-01	1.334745901224E-01	-3.967568286379E-01
H(11)	H	1.309956401294E-01	2.509003363387E-02	-4.411419123582E-01
H(13)	H	8.858708930132E-02	-7.965485622919E-02	-3.282740900889E-01
H(14)	H	1.057790406418E-01	-1.528522551607E-01	-8.248059327031E-02
H(20A)	H	8.582372724846E-02	-3.327007783585E-01	4.823114981098E-02
H(20B)	H	-1.348039068527E-01	-2.878218646953E-01	-2.377920629131E-02
H(20C)	H	-1.142429964073E-01	-3.227786828446E-01	1.828193130022E-01
